# Management of external ventricular drain: to wean or not to wean?

**DOI:** 10.1007/s00701-024-06166-z

**Published:** 2024-07-02

**Authors:** Tim Jonas Hallenberger, Thavena Tharmagulasingam, Maria Licci, Luigi Mariani, Raphael Guzman, Jehuda Soleman

**Affiliations:** 1 Department of Neurosurgery, University Hospital of Basel, Spitalstrasse 21, 4031 Basel, Switzerland; 2 Faculty of Medicine, University of Basel, Basel, Switzerland

**Keywords:** External ventricular drain, Hydrocephalus, Aneurysmatic subarachnoid hemorrhage, Traumatic brain injury, Neurosurgery

## Abstract

**Purpose:**

External ventricular drain (EVD) is one of the most frequent procedures in neurosurgery and around 15 to 30% of these patients require a permanent cerebrospinal fluid (CSF) diversion. The optimal EVD weaning strategy is still unclear. Whether gradual weaning compared to rapid closure, reduces the rate of permanent CSF diversion remains controversial. The aim of this trial is to compare the rates of permanent CSF diversion between gradual weaning and rapid closure of an EVD.

**Methods:**

This was a single-center, retrospective cohort study including patients between 2010 to 2020. Patients were divided into a weaning (WG) and non-weaning (NWG) group. The primary outcome was permanent CSF diversion rates, secondary outcomes included hospitalization time, EVD-related morbidity, and clinical outcome.

**Results:**

Out of 412 patients, 123 (29.9%) patients were excluded due to early death or palliative treatment. We registered 178 (61.6%) patients in the WG and 111 (38.4%) in the NWG. Baseline characteristics were comparable between groups. The VPS rate was comparable in both groups (NWG 37.8%; WG 39.9%, p = 0.728). EVD related infection (13.5% vs 1.8%, p < 0.001), as well as non-EVD related infection rates (2.8% vs 0%, p < 0.001), were significantly higher in the WG. Hospitalization time was significantly shorter in the NWG (WG 24.93 ± 9.50 days; NWG 23.66 ± 14.51 days, p = 0.039).

**Conclusion:**

Gradual EVD weaning does not seem to reduce the need for permanent CSF diversion, while infection rates and hospitalization time were significantly higher/longer. Therefore, direct closure should be considered in the clinical setting.

**Supplementary Information:**

The online version contains supplementary material available at 10.1007/s00701-024-06166-z.

## Introduction

The insertion of an external ventricular drain (EVD) is one of the most frequent procedures in neurosurgery. EVDs are commonly used to drain cerebrospinal fluid (CSF) or combined with an ICP pressure probe to monitor intracranial pressure (ICP) in patients. It is often an emergent procedure in critically ill patients [15, 17]. Significant impairment of CSF drainage can occur in different pathologies, including aneurysmal subarachnoid hemorrhage (aSAH), parenchymal hemorrhage, traumatic brain injury (TBI), acute hydrocephalus, intracerebral hemorrhage (ICH) with ventricular extension, infections, ischemic stroke, and neoplastic cerebral lesions [10, 26]. Despite being a frequent procedure, the ideal strategy of EVD management is still controversial. In particular, once the decision to remove the EVD is reached, the optimal weaning strategy is often ambiguous and remains elusive. One strategy is rapid closure, in which the EVD is immediately clamped and if no hydrocephalus symptoms occur removed on the following day, while another strategy is gradual weaning, in which the height of the EVD is raised step-wise over several days until clamped and removed the following day [9, 31, 33]. Both of these strategies are commonly used and could potentially harbour the risk for EVD associated complications such as infection rates and VPS placement due to delayed weaning/removal failure as well as having an impact on the length of hospital stay. Of the patients treated with EVD, 15 to 30% will need a permanent solution for CSF diversion, usually in form of a VPS [1, 33]. Whether gradual EVD weaning reduces the rates of permanent CSF diversion, as opposed to rapid EVD closure remains a matter of debate. The American Stroke Association (ASA) currently recommends a rapid closure and removal for the treatment of hydrocephalus after SAH [12]. This is also consistent with the statement of the Neurocritical Care Society, which suggested that “EVD weaning should be accomplished as quickly as is clinically feasible” [15]. Controversial to these findings, recent surveys have demonstrated that the majority of neurosurgical units still routinely perform gradual EVD weaning [4, 8, 29]. This trial aims to compare the rates of permanent CSF diversion, complications, and hospital length of stay between gradual weaning and rapid closure of EVD.

## Material and methods

Patients undergoing insertion of an EVD over a time period of 10 years (2010–2020) at the University Hospital of Basel were included in this study. Based on the management regimen the patients were divided into two groups: weaning group (WG) and non-weaning group (NWG). The chosen management regimen was based solely on the decision of the treating team, whilst mostly these decisions were made within the daily conferences including all neurosurgical staff of our department. Guidelines and rigid criteria as to which regimen should be chosen under which circumstances does not exist in our department. The decision on when to cease EVD weaning or proceed with direct closure was determined on a case-by-case basis, adhering to the following general criteria: a) removal of the EVD as soon as possible to mitigate the risk of infection, b) if vasospasms were preset, the weaning process or closure of the EVD was not initiated until the patient recovered from vasospasms, c) stable neurological state of the patient for at least 24 h before starting the weaning process or closure of the EVD and d) in case the CSF was in its appearance still bloody, the process was not initiated until the CSF cleared up. Resistance level, drainage volume of the EVD or ICP were not considered for the decision to initiate weaning/direct closure. Once the removal of the EVD was indicated in the NWG, the EVD was closed for 24 h and then, in case no hydrocephalus signs were seen clinically or on cranial computer tomography (CT), the EVD was removed. On the other hand, for the WG, the EVD was gradually weaned, by raising the height of the drain and gently decreasing the amount of CSF delivered every day until after 2–4 days of weaning, the drain was closed for 24 h and if no signs of hydrocephalus were seen clinically or on cranial CT (i.e. ventriculomegaly compared to initial CT scan), the EVD was removed. If hydrocephalus signs were seen clinically or on cranial CT a clinical decision for either CSF diversion surgery or reopening of the EVD was reached. Usually, this decision was based on the number of days the EVD was already in situ. At admission and before EVD removal a CT or magnetic resonance imaging (MRI) was concluded in all the patients.

Baseline characteristics, such as age, sex, underlying diagnosis, and comorbidities were retrospectively collected from the digital medical report system within our institution and compared between the groups. The primary endpoint was the need for permanent CSF diversion in form of a VPS after EVD removal. Endoscopic third ventriculostomy (ETV) was not undertaken in any of the patients, due to the fact that all cases were assessed to be communicating hydrocephalus, where our practice is to insert a VPS rather than offer an ETV. The secondary endpoints included hospitalization time, discharge location, EVD-related morbidity, clinical outcome (Glasgow outcome scale (GCS) categorized to 14–15, 9–13, 3–8 and modified Rankin scale (mRS) categorized to good outcome (1–3) and bad outcome (4–5)) at discharge. For pathology-dependent shunt insertion rates, the observed pathologies were grouped into: acute subarachnoid hemorrhage; trauma; infection; pathologies causing CSF obstruction (hydrocephalus, tumor, cysts); non-SAH bleedings (intraventricular hemorrhage, intracerebral hemorrhage, postoperative hemorrhage, AVM bleeding).

All statistical analyses were done using SPSS Version 28.0.1.0 (IBM Corp., Armonk, NY). Descriptive statistics were performed for all outcome variables. Contingency tests were done using the Chi-square or Fisher exact test, while for continuous variables the Mann–Whitney U test was used. In addition, Kaplan Meyer curves with a log-rank test were concluded for the primary outcome. A p value of < 0.05 was considered significant.

## Results

### Baseline characteristics

**Fig. 1:**
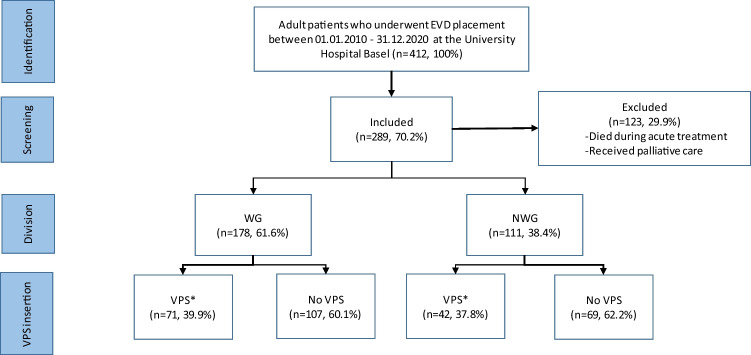
Flow-chart of the enrolled patients

Out of 412 consecutive patients undergoing insertion of an EVD, 289 (70.2%) patients were included in the retrospective analysis. We excluded 123 (29.9%) patients which died during the acute treatment or who received palliative treatment due to the underlying disease. In patients where palliative treatment was initiated, no weaning of the EVD was undertaken, but rather the EVD removed without further treatment, even if hydrocephalus occurred at a later phase. The WG consisted of 178 (61.6%) patients and the NWG of 111 (38.4%) patients (Fig. 1). Baseline characteristics are depicted in Table 1. The cohort included 137 (47.4%) females, with a median age of 58 years (IQR 45–68 years). The most frequent underlying diagnosis was aneurysmatic subarachnoid hemorrhage (aSAH, 41.2%), followed by intracerebral hemorrhage (17.0%), traumatic brain injury (TBI, 14.5%), other indications (5.2%), tumor (4.8%), isolated intraventricular hemorrhage (IVH, 4.2%), post-infectious (3.8%), and ischemic stroke and arteriovenous malformation (AVM) (3.1% each). ASAH was significantly more common in the weaning group (p = 0.017). Median time from EVD insertion to EVD removal were 5 days (IQR 2.5 – 10) in the NWG and 7 days (IQR 5 – 11) in the WG (p < 0.001). American Society of Anesthesiology score (ASA-score) and Charlson Comorbidity Index (CCI) were comparable between the two groups (Table 1 and supplemental Table 2).

**Table 1:** Baseline Characteristics

Parameter		Non-Weaning Group (n = 111)		Weaning Group (n = 178)		Total (n = 289)		P- value
Age (years)		56	48–69	58	44–67	58	45–68	0.437
Gender	**male**	63	50.0%	89	50.0%	152	52.6	0.263
Diagnosis	**aSAH**	36	32.4%	83	46.6%	119	41.2	0.017
	**Trauma**	18	16.2%	24	13.5%	42	14.5	0.521
	**Acute Hydrocephalus**	3	2.7%	1	0.6%	4	1.4	0.160
	**IVH (non-aneurysmatic)**	6	5.4%	6	3.4%	12	4.2	0.339
	**Infection**	3	2.7%	8	4.5%	11	3.8	0.540
	**Ischemic Stroke**	5	4.5%	4	2.2%	9	3.1	0.312
	**Intracerebral haemorrhage**	21	18.9%	28	15.7%	49	17.0	0.482
	**Tumor**	7	6.3%	7	3.9%	14	4.8	0.405
	**Postoperative bleeding**	3	2.7%	0	0.0%	3	1.0	0.056
	**AVM**	2	1.8%	7	3.9%	9	3.1	0.490
	**Colloid cyst**	1	0.9%	1	0.6%	2	0.7	1.00
	**Other**	6	5.4%	9	5.1%	15	5.2	0.896
GCS preoperatively	**14–15**	25	22.5%	44	24.7%	69	23.9	0.604
	**9–13**	28	25.2%	36	20.2%	64	22.1	
	**3–8**	58	52.3%	98	55.1%	156	54.0	
mRS preoperatively	**1–3**	81	73.1%	132	74.2%	213	73.7	0.824
	**4–5**	30	27.0%	46	25.8%	76	26.3	
Time of indwelling EVD (days)		5	2.5–10	7	5–11	7	4–11	< 0.001
Time to EVD (days)		0	0–1	0	0–1	0	0–1	0.203

*aSAH* = *aneurysmatic subarachnoid haemorrhage, IVH* = *intraventricular haemorrhage, AVM* = *arterio-venous malformation, GCS* = *Glasgow coma scale, mRS* = *modified rankin scale, EVD* = *external ventricular drain*

*All continuous values presented as mean* ± *standard deviation*

*All nominal values presented as N and %*

### Primary outcome

**Fig. 2:**
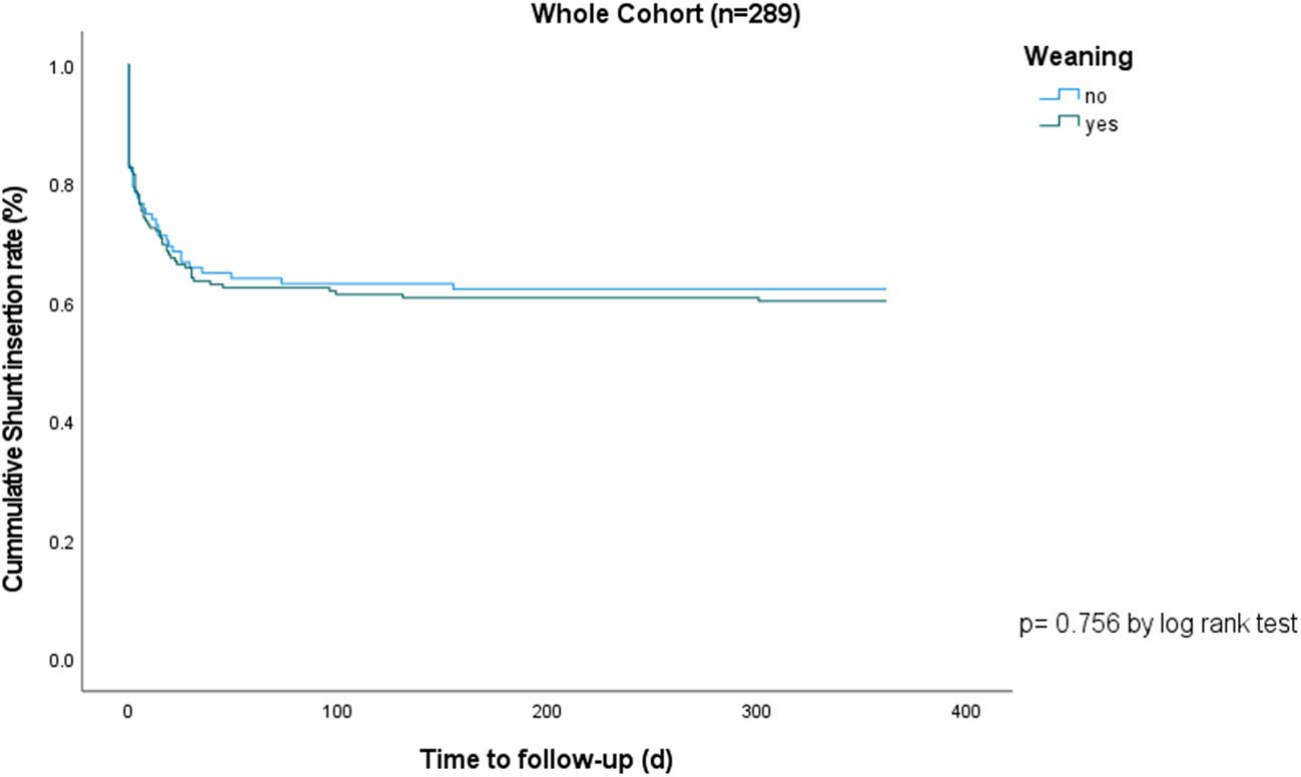
Cumulative shunt insertion rate after EVD removal (day zero) over the one-year follow-up period

**Fig. 3:**
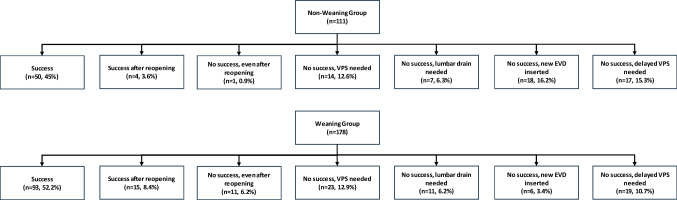
Flow-chart of the case distribution for the specific outcomes defined in the weaning- and non-weaning group

The rate of VPS insertion after EVD removal was comparable between the two groups, with a rate of 37.8% in the NWG and 39.9% in the WG (p = 0.728, OR 1.09 [95%CI 0.67 – 1.77], Table 2) and remained non-significant in both groups over the whole observation period (log-rank test x^2^ = 0.096, mean time to shunt for WG 225.8 ± 12.95 days [95%CI 200.5–251.2 days] vs. mean time to shunt for NWG 231.7 ± 16.3 days [95%CI 199.8 – 263.6 days], p = 0.756, Fig. 2). When stratified for etiology, no significant difference between the WG and NWG was seen across all strata (Supplemental Table 1). Multivariable regression analysis showed no potential factors significantly influencing VPS insertion rates (results not shown). Of those patients where EVD closure initially failed in 12.8% a VPS was inserted immediately, in 8.3% an EVD was re-inserted, in 6.2% a lumbar drain was inserted, while in 6.6% after reopening the EVD for a couple of days the EVD could be removed, however in 4.2% a VPS was still needed after reopening the EVD (Fig. 3). In 12.5% of the patients, where EVD removal was initially successful, a delayed VPS insertion was needed due to chronic hydrocephalus. In the WG, VPS was significantly more often needed after reopening of the EVD (6.2% vs 0.9%, p = 0.003), while a new EVD was re-inserted significantly more often, without influencing VPS rate, in the NWG (16.2% vs 3.4%, p < 0.001, Table 2). The reasons for unsuccessful weaning or closure were progression of symptoms (49.6%), progression of symptoms and ventricular enlargement on CT (24.4%), isolated ventricular enlargement on CT (13.0%), and CSF leak with or without ventricular enlargement and/or symptoms (4.9% and 3.3% respectively, Table 2). Progression of symptoms was significantly more common in the NWG (66.1% vs 35.8%, p < 0.001) while ventricular enlargement without symptoms was significantly more common in the WG (22.4% vs 1.8%, p < 0.001). Overall shunt insertion rate was significantly higher in aSAH and infection groups (p < 0.001 and p = 0.038 respectively). Further, looking at the aSAH group separately, VPS insertion occurred significantly more often in the WG than in the NWG (p < 0.001). Time from EVD closure or removal to VPS was significantly shorter in the WG (2.68 ± 4.50 days without delayed weaning failure (VPS insertion) patients (n = 22), 46.27 ± 65.62 days with delayed weaning failure) than in the NWG (12.74 days ± 27.16 days, p = 0.028, Table 2).

**Table 2:** Primary and Secondary Outcomes

Parameter		Non-Weaning Group (n = 111)		Weaning Group (n = 178)		Total (n = 289)		P value
Insertion of VPS after EVD removal		42	37.8	71	39.9	113	39.1	0.728
Initial weaning success	**yes**	50	45.0	93	52.2	143	49.5	0.234
	yes, after reopening*	4	3.6	15	8.4	19	6.6	0.144
	**No, even after reopening**	1	0.9	11	6.2	12	4.2	0.033
	**No, VPS needed**	14	12.6	23	12.9	37	12.8	0.939
	**lumbar drain†**	7	6.3	11	6.2	18	6.2	0.965
	**new EVD inserted‡**	18	16.2	6	3.4	24	8.3	< 0.001
	**VPS delayed needed**	17	15.3	19	10.7	36	12.5	0.245
Reason for unsuccessful weaning	**CSF leak**	1	1.8	5	7.5	6	4.9	0.219
	**Progression of symptoms**	37	66.1	24	35.8	61	49.6	< 0.001
	**Ventricle enlargement, without symptoms**	1	1.8	15	22.4	16	13.0	< 0.001
	**CSF leak and ventricle enlargement**	3	5.4	3	4.5	6	4.9	1.000
	**CSF leak and symptoms**	2	3.6	2	3.0	4	3.3	1.000
	**Symptoms and ventricular enlargement**	12	21.4	18	26.9	30	24.4	0.484
Amount of weaning attempts	**one**	104	93.7	142	79.8	246	85.1	0.001
	**two**	6	5.4	33	18.5	39	13.5	0.001
	**three**	1	0.9	3	1.7	4	1.4	1.000
Time to VPS§		12.74	27.158	2.68	4.497	14.87	36.706	0.028
EVD associated morbidity		28	25.2	63	35.4	91	31.5	0.070
Infection	**no**	109	98.2	149	83.7	258	89.3	< 0.001
	**yes EVD related**	2	1.8	24	13.5	26	9.0	< 0.001
	**yes non-EVD related**	0	0	5	2.8	5	1.7	0.160
EVD dislocation		5	4.5	4	2.2	9	3.1	0.283
EVD related bleeding		3	2.7	11	6.2	14	4.8	0.181
Hospitalisation time (days)		23.66	14.507	24.93	9.497	24.44	11.673	0.039
GCS at discharge	**14–15**	73	74.5	122	70.9	195	72.2	0.751
	**9–13**	20	20.4	42	24.4	62	23.0	
	**3–8**	5	5.1	8	4.7	13	4.8	
mRS at discharge	**1–3**	63	64.3	100	58.1	163	60.4	0.083
	**4–5**	33	33.7	72	41.9	105	38.9	

*VPS* = *ventriculoperitoneal shunt, EVD* = *external ventricular drain, CSF* = *cerebro spinal fluid, GCS* = *Glasgow outcome scale, mRS* = *modified rankin scale, aSDH* = *acute subdural hematoma, aEDH* = *acute epidural hematoma, IVH* = *intraventricular hemorrhage, ICH* = *intracerebral hemorrhage*

*All continuous values presented as mean* ± *standard deviation*

*All nominal values presented as N and %*

^*^ Meaning the EVD was reopened and patients were assessed for 48–72 days before reattempting weaning in the WG or direct closure in the NWG. Three patients (1 WG, 2 NWG) became shunt dependent after initially successful weaning/removal

^*†*^*Lumbar drain was inserted to alleviate progressive symptoms and CSF leakage additional to the indwelling EVD during weaning or shortly after direct closure (NWG). Patients were reassessed for a reattempt in weaning/direct closure after 48–72 h). Nine patients (6 WG, 3 NWG) became shunt dependent after initially successful weaning/removal*

^*‡*^*New EVD was inserted in patients with new-onset symptoms after initial weaning/direct closure (*< *7 days after initial EVD insertion ≠ delayed failure) and in EVD infection or dysfunction. Patients were thereafter re-assessed for EVD removal and treated as initially planned (WG or NWG). Seven patients (4 WG, 3 NWG) became shunt dependent after initially successful weaning/removal*

^*§*^*without patients with delayed weaning failure (delayed VPS insertion) (n* = *22)*

### Secondary outcomes

EVD related infection rate in the WG (13.5%) was significantly higher than in the NWG (1.8%, p < 0.001). Similarly, non-EVD related infection rate was significantly higher in the WG (2.8%) than in the NWG (0%, p < 0.001, Table 2). Hospitalization time was significantly shorter in the NWG compared to the WG (WG 24.93 ± 9.50 days and NWG 23.66 ± 14.51 days, p = 0.039). No significant difference between the overall hospitalization time regarding EVD related infections in the NWG and WG was seen (WG 24 days (IQR 19–30); NWG 27.5 days). Overall EVD–associated morbidity (WG 35.4% and NWG 25.2%, p = 0.070), dislocation rate of the EVD (WG 2.2% and NWG 4.5%, p = 0.283), bleeding rates (WG 6.2% and NWG 2.7%, p = 0.181) and type of bleeding showed no significant difference between the groups. Further, discharge location, as well as clinical outcome at release did not differ between the groups either (Table 2).

## Discussion

### Summary of the main findings

This retrospective trial aimed to evaluate the benefits and harms of direct closure (NWG) versus gradual weaning (WG) of an EVD. The optimal management of EVD in the context of various underlying causes is controversial. In our study, we found that the rate of VPS placement was comparable between the two groups. Further, the rate of EVD related and non-EVD related infection rate was significantly higher in the WG, while the hospitalization time was significantly shorter in the NWG.

### VPS insertion rate

We found that the VPS insertion rate was comparable and did not differ significantly between the NWG and the WG. This is consistent with the findings of Klopfenstein et al., who compared rapid and gradual weaning of EVD in 81 patients after aSAH in a randomized controlled fashion [23]. They found no benefit of gradual weaning in preventing a VPS placement and therefore prompt closure is recommended by the authors to reduce length of ICU and hospital stay [23]. However, the study showed clear limitations, since only one weaning attempt was tolerated before VPS was placed, and the study was of low quality, leading to insufficient support for either strategy of EVD discontinuation [5, 29]. Likewise, a recent systematic review with meta-analysis by Palasz et al. found no significant difference in VPS insertion rate between direct closure and gradual weaning. However, due to the limited data available and the fact that most studies included were of retrospective design, the results by Palasz et al. should be interpreted with care [29]. Contrarily, a retrospective trial by Jabberli et al. compared two institutions using different weaning strategies in a total of 965 patients after SAH. The authors concluded that there was a significant independent association between direct closure strategy and VPS placement. Simultaneously, patients treated with gradual weaning had a higher delayed VPS rate, but without an increased risk of infection despite longer EVD treatment [20]. Chung et al. reported reduced VPS insertion rates with prompt closure additionally to shorter hospital stay and decreased EVD-associated complications [11]. These findings were consistent with those of Rao et al., showing a significant reduction in VPS insertion rate in patients with direct closure after aSAH [31]. Our results support the results of Klopfenstein and Palasz, where neither treatment regime was superior to the other in terms of VPS insertion rate [23, 29]. A recent meta-analysis showed, that gradual and rapid EVD weaning lead to comparable VPS insertion and EVD related infection rates. However, rapid EVD weaning leads to significantly shorter ICU stays, shorter hospital stays and should thus be preferred over gradual weaning [14]. Despite the overall controversial results described in the literature, most papers are based on patients treated with EVD after aSAH. There are currently no studies comparing different EVD discontinuation schemes in a wide variety of underlying pathologies and thus it remains elusive whether direct closure or gradual weaning should be preferred with other underlying pathologies. Nwachuku et al. compared intermittent versus continuous EVD drainage in adult patients with severe TBI [27]. Although different in design, intermittent drainage could be interpreted as direct closure with repeated clamping. No differences were found in the rates of VPS placement, ICU stay, and CSF infections [7, 27]. Our findings highlight the importance of aSAH (most common indication for EVD in our cohort) as cause for VPS insertion rates, as patients with aSAH and weaning regime showed significantly higher shunt rates than in the non-weaning group. However, the effect of other, different underlying pathologies on the shunt insertion rate based on the EVD treatment scheme remains unknown. Interestingly, when considering the reasons for unsuccessful weaning or closure, we found that progression of symptoms was significantly more common in the NWG while ventricular enlargement without symptoms was significantly more common in the WG. The reason for shunting in patients without new symptoms was mostly due to the development of a radiologically progressive ventriculomegaly, seen progressive on more than one follow-up CT. The second most common reason was the presence of a large amount of residual blood in form of IVH and or ICB in imaging resulting in ventricular enlargement. Based on the present results, gradual weaning does not seem to hold a benefit over direct closure in different pathologies, while also the amount of clamping attempts prior to EVD removal appears to have only limited influence on the rate of VPS insertion [3]. Currently, an international multicenter trial (DRAIN Trial, NCT03948256) is ongoing, investigating whether direct closure or gradual EVD weaning in aSAH patients contributes to VPS insertion, mortality and EVD infection rates [6].

### Hospitalization rate, infection rates and clinical outcome

We found the hospitalization time to be significantly shorter in the NWG compared to the WG. This differs from the findings of Chung et al., who reported no significant difference in the hospitalization period [11]. Another study, which analyzed continuous versus intermittent EVD drainage also found no difference in length of stay [2]. However, the majority of the literature reports, that gradual weaning is associated with a prolonged ICU stay and overall longer hospitalization time [9, 20, 23, 29, 31]. To note, our cohort consists of various pathologies which could have affected the general length of hospitalization. However, both groups were evenly matched regarding the etiology, which therefore should not affect the shown difference between the two groups [22].In the current study, we found that EVD related infection rates and non-EVD related infection rates were significantly higher in the WG. Namely, the infection rate in the WG was nearly 14 times higher than in the NWG. The length of hospitalization could have played a role in the development of these secondary infections, yet the hospitalization time did not differ regarding EVD related infections between groups. Hagel et al. described, that patients with EVD-associated infections have significantly more often other concurrent healthcare-associated infections, which might be a consequence of the prolonged length of stay in the ICU [16]. This could explain the significant increase, especially in non-EVD-related infections, within the current study. Regarding EVD-related infections, there are a few studies that have looked more closely at the duration of EVD placement affecting the infection rate. Mostly they compared patients with and without ventriculitis after EVD treatment and found that the incidence of ventriculitis was significantly higher in patients with longer EVD duration and longer hospital stay [19, 21, 22]. Similarly, Dos Santos et al. found EVD in place more than 10 days has significantly higher odds of infections than less than 10 days [13]. In the current study, the overall median EVD duration in place was around between 5 and 7 days in the groups with the WG having the shorter duration. This risk for EVD-related infections could also be increased by the higher likelihood of contamination when the EVD is kept open during the weaning process. A randomized trial by Rao et al., comparing intermittent/rapid versus continuous/gradual EVD approach, was stopped early due to the significantly higher rates of ventriculitis and nonpatent EVD in the continuous/gradual EVD group [31]. Similarly, Olson et al. found that a clinically relevant percentage of patients with continuous CSF drainage (17.6% vs 3.8%, p = 0.132) was associated with a higher incidence of ventriculitis compared to closed EVD management with intermittent drainage [28]. Another reason for the increased infection rate could be explained by higher probability of EVD manipulations in the WG. Multiple studies have shown that reducing CSF sampling and EVD manipulations lead to lower infection rates [19, 24, 25, 35]. When looking at studies, directly comparing different EVD managements, two of them described no significant difference in infection rate [20, 27]. The meta-analysis by Palasz et al. found that the risk of infection was significantly higher for patients undergoing continuous CSF drainage compared to intermittent drainage. However, they found no significant difference in infection rate comparing rapid and gradual EVD weaning [29]. Further, in terms of preventing EVD-related infections the use of Bactiseal EVD might be beneficial, however when the infection is already present, even the use of antibiotic-coated EVD show no advantage over regular EVD [34]. Further studies described that EVD-related infections could be prevented with chlorhexidine dressings [32, 36]. Apart from these findings, a recent systematic review found, that there is a lot of controversies regarding the prevention and treatment of ventriculitis, which after all these years still leads to unclear practice when it comes to preventing EVD infections [30]. To note, these infections also have a substantial effect on clinical morbidity, mortality and healthcare costs [18]. Regarding overall EVD related morbidity, in the current study we did not observe a significant difference in morbidity**.** EVD dislocation rate, which is often referred to as non-functioning or non-patent EVD, is reported with various definitions throughout the literature. Rao et al. found a lower incidence of non-functioning EVDs (blocked EVD or reinsertion of the EVD) in the direct closure group without reaching statistical significance [31]. Kim et al. showed a higher rate of EVD complications in the gradual weaning group, including clogged/blocked EVDs and self-removal of the EVD [21]. Olson et al., who compared continuous drainage with intermittent drainage, found that patients with continuous drainage had a significantly higher risk of developing a nonpatent EVD (i.e. EVD needing a flushing or new EVD insertion) [28]. Contrary to these findings, we found in the current study that patients in the NWG, had a slightly higher trend for EVD dislocation, although our result did not reach statistical significance. Further, we found no difference amongst the groups regarding the clinical outcome of the patients, measured by mRS, and the destination at discharge. These finding are in agreement with two other studies, that describe no difference in the clinical outcome at discharge measured by mRS [20, 22].

### Limitations and strengths

This retrospective study is subject to all the limitations of data collection inherent in such work. The decision of the weaning regime (WG/NWG), the timing when weaning should be initiated, when to repeat a clamp trial, or when to insert a VPS was made by the treating team and is therefore heterogeneous and did not follow predefined criteria, but rather a clinical case by case decision. Some of the variables included were evaluated through the judgement or measurements of individual investigators, which may vary in accuracy or consistency increasing the variance of our results. The procedures (EVD or VPS insertions) were performed by different surgeons and thus a certain variability in the decision-making for shunt insertion was present. Other factors, such as CSF sampling values, underlying etiology, amount of drained CSF, steps of weaning needed before clamping the EVD, were not part of the decision making in the clinical setting and therefore were not assessed and analysed. Clearly, these factors could have influenced the outcome within the groups as well. Including different pathologies and compiling them into one data set contributes to the heterogeneity of the results and despite stratifying for etiology, are limiting the overall generalizability of this work. Due to the retrospective design of this work, some analyses were limited due to missing data and sample size issues and as such, long- term functional outcome and infection data were not available, limiting those conclusions beyond the hospitalization.

The strengths of our trial are the wide range of pathologies covered, which increases external validity as well as the rather large sample size included in this trial.

## Conclusion

Based on our results, gradual weaning of EVD does not seem to have an advantage over direct closure in terms of the need for permanent CSF diversion surgery. However, due to the limitations of this study, results should be interpreted with care. Gradual weaning seems to be associated with a higher risk of infections, and a longer hospitalization time. Therefore, direct EVD closure should be considered as an option in the clinical setting. Future randomized trials are eagerly awaited to confirm our results.

## Supplementary Information

Below is the link to the electronic supplementary material.

Supplementary file1 (DOCX 36 KB)

## Abbreviations

aSAH: Aneurysmal Subarachnoid Haemorrhage
EVD: External Ventricular Drain
NWG: Non-weaning group
VPS: Ventriculoperitoneal Shunt
WG: Weaning group

## Declarations

### Data sharing agreement

An anonymized data set will be made available by the corresponding author upon reasonable request.
