# Supplementary material for: Management of external ventricular drain: to wean or not to wean?
Source: Acta Neurochir (Wien). 2024 Jul 2;166(1):279. doi: 10.1007/s00701-024-06166-z (PMC11219415; doi:10.1007/s00701-024-06166-z)
Supplement: Supplementary file 1 — Supplementary file1 (DOCX 36 KB) [file 701_2024_6166_MOESM1_ESM.docx]

**Supplemental Material**

**Hallenberger et al.**

**Table of contents**

[**Supplemental Table 1:** Stratified Results for the primary outcome across the different aetiologies 3](#_Toc165642020)

[**Supplemental Table 4:** Additional baseline data 4](#_Toc165642021)

# **Supplemental Table 1:** Stratified Results for the primary outcome across the different aetiologies

| **Variable** | **Weaning group/ p** | **Rate of VPS insertion** | **Time to VPS** | **EVD related infection** | **EVD related bleeding** | **Hospitalization time (days)** |
| --- | --- | --- | --- | --- | --- | --- |
| **aSAH (n=119)** | **NWG (n=36, 30%)** | 16 (13.4) | 6.37 ± 8.34 | 1 (0.8) | 1 (0.8) | 22.64 ± 10.8 |
|  | **WG (n=83, 70%)** | 44 (37.0) | 2.61 ± 4.39 | 12 (10.1) | 7 (5.9) | 24.94 ± 8.6 |
|  | **p-value** | 0.391 | 0.080 | 0.106 | 0.432 | **0.041** |
| **Trauma (n=42)** | **NWG (n=18, 43%)** | 8 (19.0) | 6 (14.3) | 0 | 0 | 27.33 ± 11.94 |
|  | **WG (n=24, 57%)** | 7 (16.7) | 18 (42.9) | 3 (7.1) | 0 | 26.00 ± 10.23 |
|  | **p-value** | 0.307 | **0.007** | 0.247 | - | 0.282 |
| **Acute HCP (n=4)** | **NWG (n=3, 75%)** | 8 (19.0) | 0 | 0 | 0 | 20.67 ± 8.51 |
|  | **WG (n=1, 25%)** | 7 (16.7) | 0 | 0 | 0 | 8.0 |
|  | **p-value** | 0.307 | - | - | - | 0.180 |
| **IVH (n=12)** | **NWG (n=6, 50%)** | 3 (25.0) | 2.67 ± 4.62 | 0 | 0 | 17.5 ± 3.67 |
|  | **WG (n=6, 50%)** | 2 (16.7) | 4.00 ± 1.41 | 0 | 0 | 23.33 ± 7.94 |
|  | **p-value** | 1.000 | 0.554 | - | - | 0.147 |
| **Infection (n=11)** | **NWG (n=3, 28%)** | 0 | - | 0 | 0 | 28.0 ± 12.12 |
|  | **WG (n=8, 72%)** | 1 (9.1) | - | 3 (27.3) | 0 | 27.38 ± 9.27 |
|  | **p-value** | 1.000 | - | 0.491 | - | 0.758 |
| **Intracerebral haemorrhage (n=49)** | **NWG (n=21, 43%)** | 3 (6.1) | 1.67 ± 2.89 | 1 (2.0) | 1 (2.0) | 18.30 ± 11.43 |
|  | **WG (n=28, 57%)** | 9 (18.4) | 3.80 ± 4.76 | 4 (8.2) | 1 (2.0) | 22.96 ± 9.24 |
|  | **p-value** | 0.192 | 0.608 | 0.376 | 1.000 | 0.056 |
| **Ischemic Stroke (n=9)** | **NWG (n=5, 56%)** | 1 (11.1) | 49.00 | 0 | 0 | 24.00 ± 14.02 |
|  | **WG (n=4, 44%)** | 1 (11.1) | 0 | 0 | 0 | 19.75 ± 2.99 |
|  | **p-value** | 1.000 | 0.317 | - | - | 0.902 |
| **Tumor (n=14)** | **NWG (n=7, 50%)** | 3 (14.3) | 6.00 ± 7.21 | 0 | 0 | 48.71 ± 31.11 |
|  | **WG (n=7, 50%)** | 1 (7.1 | 0 | 0 | 2 (14.3) | 22.86 ± 6.54 |
|  | **p-value** | 0.559 | 0.346 | - | 0.462 | 0.180 |
| **Postoperative bleed (n=3)** | **NWG (n=3, 100%)** | 2 (66.7) | 18.5 ±23.34 | - | - | 23.33 ± 9.87 |
|  | **WG (n=0)** | - | - | - | - | - |
|  | **p-value** | - | - | - | - | - |
| **AVM (n=9)** | **NWG (n=2, 22%)** | 1 (11.1) | 0 | 0 | 1 (11.1) | 20.5 ± 13.44 |
|  | **WG (n=7, 78%)** | 2 (22.2) | 0 | 1 (11.1) | 1 (11.1) | 33.29 ± 14.27 |
|  | **p-value** | 1.000 | 1.000 | 1.000 | 0.417 | 0.143 |
| **Colloid Cyst (n=2)** | **NWG (n=1, 50%)** | 0 | - | 0 | 0 | 27 |
|  | **WG (n=1, 50%)** | 0 | - | 0 | 0 | 22 |
|  | **p-value** | - | - | - | - | 0.317 |
| **Other* (n=15)** | **NWG (n=6, 40%)** | 3 (20.0) | 0 | 0 | 0 | 13.33 ± 6.59 |
|  | **WG (n=9, 60%)** | 4 (26.7) | 5.25 ± 7.27 | 1 (6.7) | 0 | 26.78 ± 14.13 |
|  | **p-value** | 1.000 | 0.079 | 1.000 | - | **0.033** |

*Abbreviations: aSAH= aneurysmatic subarachnoid haemorrhage, IVH= intraventricular haemorrhage, AVM= arterio-venous malformation, NWG= Non-weaning group, WG= weaning group, VPS=Ventriculoperitoneal Shunt, EVD= external ventricular drain*

*All continuous values presented as mean ± standard deviation*

**13 angio-negative subarachnoid haemorrhages and 2 traumatic intracerebral haemorrhages with ventricular affection*

*All nominal values presented as N and %*

# **Supplemental Table 4:** Additional baseline data

|  | | **Non-Weaning Group**  **(n=111)** | | **Weaning Group**  **(n=178)** | | **Total**  **(n=289)** | | **P- value** |
| --- | --- | --- | --- | --- | --- | --- | --- | --- |
|  |  |  |  |  |  |  |  |  |
| Motor deficits | | 13 | 11.7% | 20 | 11.2% | 33 | 11.4 | 0.902 |
| Sensory deficits | | 10 | 9.0% | 22 | 12.4% | 32 | 11.1 | 0.377 |
| Headache | | 48 | 43.2% | 78 | 43.8% | 126 | 43.6 | 0.923 |
| Gait disturbance | | 2 | 1.8% | 5 | 2.8% | 7 | 2.4 | 0.711 |
| Seizures | | 7 | 6.3% | 24 | 13.5% | 31 | 10.7 | 0.055 |
| Speech disorder | | 5 | 4.5% | 12 | 6.7% | 17 | 5.9 | 0.608 |
| Confusion | | 5 | 4.5% | 11 | 6.2% | 16 | 5.5 | 0.608 |
| Vertigo | | 14 | 12.6% | 17 | 9.6% | 31 | 10.7 | 0.413 |
| Cognitive decline | | 1 | 0.9% | 3 | 1.7% | 4 | 1.4 | 1.000 |
| Nausea/Emesis | | 20 | 18.0% | 28 | 15.7% | 48 | 16.6 | 0.611 |
| Other symptoms | | 7 | 6.3% | 14 | 7.9% | 21 | 7.3 | 0.620 |
| Bloodthinners | | 26 | 23.4% | 59 | 33.3% | 85 | 29.5 | 0.073 |
| **ASA score** | **1** | 1 | 1.1% | 1 | 0.7% | 2 | 0.7 | 0.690 |
|  | **2** | 5 | 5.3% | 16 | 10.7% | 21 | 7.3 |  |
|  | **3** | 39 | 41.5% | 67 | 44.7% | 106 | 36.7 |  |
|  | **4** | 43 | 45.7% | 59 | 39.3% | 102 | 35.3 |  |
|  | **5** | 5 | 5.3% | 6 | 4.0% | 11 | 3.8 |  |
|  | **6** | 1 | 1.1% | 1 | 0.7% | 2 | 0.7 |  |
| **CCI** | **0** | 31 | 27.9% | 44 | 24.7% | 75 | 26.0 | 0.300 |
|  | **1** | 17 | 15.3% | 35 | 19.7% | 52 | 18.0 |  |
|  | **2** | 14 | 12.6% | 40 | 22.5% | 54 | 18.7 |  |
|  | **3** | 21 | 18.9% | 25 | 14.0% | 46 | 15.9 |  |
|  | **4** | 10 | 9.0% | 15 | 8.4% | 25 | 8.7 |  |
|  | **5** | 6 | 5.4% | 10 | 5.6% | 16 | 5.5 |  |
|  | **6** | 6 | 5.4% | 3 | 1.7% | 9 | 3.1 |  |
|  | **7** | 1 | 0.9% | 3 | 1.7% | 4 | 1.4 |  |
|  | **8** | 2 | 1.8% | 2 | 1.1% | 4 | 1.4 |  |
|  | **9** | 2 | 1.8% | 1 | 0.6% | 3 | 1.0 |  |
|  | **10** | 1 | 0.9% | 0 | 0.0% | 1 | 0.3 |  |
| Hypertension | | 39 | 35.1% | 55 | 30.9% | 94 | 32.5 | 0.455 |
| Coronary disease | | 11 | 9.9% | 19 | 10.7% | 30 | 10.4 | 0.160 |
| Cardiac stent | | 6 | 5.4% | 9 | 5.1% | 15 | 5.2 | 0.896 |
| Cardiac bypass surgery | | 3 | 2.7% | 1 | 0.6% | 4 | 1.4 | 0.130 |
| Diabetes | | 22 | 19.8% | 25 | 14.0% | 47 | 16.3 | 0.196 |
| Peripheral artery disease | | 2 | 1.8% | 8 | 4.5% | 10 | 3.5 | 0.327 |
| Carotid stent | | 1 | 0.9% | 4 | 2.2% | 5 | 1.7 | 0.652 |
| Atrial fibrillation | | 10 | 9.0% | 22 | 12.4% | 32 | 11.1 | 0.377 |
| Cholesterin | | 4 | 3.6% | 5 | 2.8% | 9 | 3.1 | 0.737 |
| Hematological disease | | 4 | 3.6% | 5 | 2.8% | 9 | 3.1 | 0.737 |
| Liver failure | | 0 | 0.0% | 1 | 0.6% | 1 | 0.3 | 1.000 |
| Renal failure | | 9 | 8.1% | 11 | 6.2% | 20 | 6.9 | 0.530 |
| Nicotine abuse | | 15 | 13.5% | 20 | 11.2% | 35 | 12.1 | 0.564 |
| Alcohol abuse | | 11 | 9.9% | 10 | 5.6% | 21 | 7.3 | 0.172 |
| Epilepsy | | 4 | 3.6% | 6 | 3.4% | 10 | 3.5 | 1.000 |
| Adipositas | | 8 | 7.2% | 6 | 3.4% | 14 | 4.8 | 0.140 |
| Other Comorbidity | | 45 | 40.5% | 75 | 42.1% | 120 | 41.5 | 0.789 |

*Abbreviations: ASA=American Society of Anethesiologists Physical Status Classification System, CCI= Charleston Comorbidity Index*

*All continuous values presented as mean ± standard deviation*

*All nominal values presented as N and %*
